# Crystal structure of (15,20-bis­(2,3,4,5,6-penta­fluoro­phen­yl)-5,10-{(4-methyl­pyridine-3,5-di­yl)bis­[(sulfanediyl­methyl­ene)[1,1′-biphen­yl]-4′,2-di­yl]}porphyrinato)nickel(II) di­chloro­methane *x*-solvate (*x* > 1/2)

**DOI:** 10.1107/S2056989019012453

**Published:** 2019-09-27

**Authors:** Florian Gutzeit, Tjorge Neumann, Christian Näther, Rainer Herges

**Affiliations:** a Otto-Diels-Institut für Organische Chemie, Christian-Albrechts-Universität Kiel, Otto-Hahn-Platz 4, D-24098 Kiel, Germany; b Institut für Anorganische Chemie, Christian-Albrechts-Universität Kiel, Max-Eyth Str. 2, D-24118 Kiel, Germany

**Keywords:** crystal structure, nickel porphyrin, square-pyramidal Ni^II^ coordination, C—H⋯F hydrogen bonding, solvate

## Abstract

The title compound, [Ni(C_64_H_33_F_10_N_5_S_2_)]·*x*CH_2_Cl_2_, consists of discrete Ni^II^ porphyrin complexes, in which the five-coordinate Ni^II^ cations are in a distorted square-pyramidal coordination geometry. The four porphyrin nitro­gen atoms are located in the basal plane of the pyramid, whereas the pyridine N atom is in the apical position. The porphyrin plane is strongly distorted and the Ni^II^ cation is located above this plane by 0.241 (3) Å and shifted in the direction of the coordinating pyridine nitro­gen atom. The pyridine ring is not perpendicular to the N_4_ plane of the porphyrin moiety, as observed for related compounds. In the crystal, the complexes are linked *via* weak C—H⋯F hydrogen bonds into zigzag chains propagating in the [001] direction. Within this arrangement cavities are formed, in which highly disordered di­chloro­methane solvate mol­ecules are located. No reasonable structural model could be found to describe this disorder and therefore the contribution of the solvent to the electron density was removed using the SQUEEZE option in *PLATON* [Spek (2015 ▸). *Acta Cryst*. C**71**, 9–18].

## Abstract 2

The title compound consists of discrete complexes with a square-pyramidal NiN_5_ coordination polyhedron for the metal ions. The complexes are linked by C—H⋯F hydrogen bonds into chains propagating along [001].

## Chemical context   

**Figure 1:**
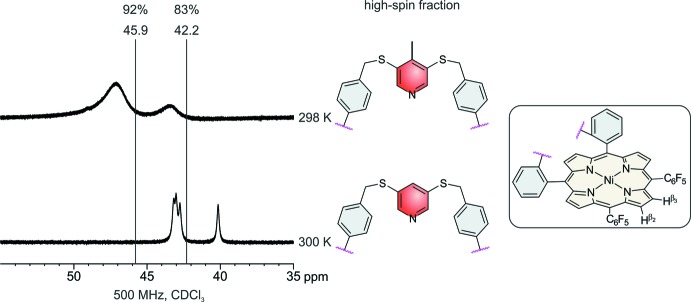
Comparison of the paramagnetic shifts of the β-pyrrole H atoms of the parent compound and the title compound, indicating increased intra­molecular coordination.

Ni^II^ porphyrins are emerging in a number of applications including photoswitchable MRI contrast agents (Venkataramani *et al.*, 2011 ▸; Dommaschk *et al.*, 2014 ▸, 2015*a* ▸,*b* ▸), redox catalysts (Eom *et al.*, 1997 ▸; Han *et al.*, 2015 ▸) or catalysts in the hydrogen evolution reaction (HER) (Han *et al.*, 2016 ▸; Solis *et al.*, 2016 ▸; Maher *et al.*, 2019 ▸). The axial coordination of Ni^II^ porphyrins has been studied extensively regarding the underlying equlibria (Caughey *et al.*, 1962 ▸; McLees & Caughey, 1968 ▸; Walker *et al.* 1975 ▸), conformational changes (Jia *et al.*, 1998 ▸) and photo-induced complex formation and dissociation (Kim *et al.*, 1983 ▸; Kim & Holten, 1983 ▸). Moreover, the axial coordination determines the spin state of these complexes **(**Renner *et al.*, 1991 ▸; Jentzen *et al.*, 1995 ▸). Upon coordination of one axial ligand, Ni^II^ porphyrins undergo spin transition from a diamagnetic (*S* = 0) square-planar, low-spin (LS) state with a coordination number (CN) of four (CN4) to a paramagnetic (*S* = 1), square-pyramidal (CN5), high-spin (HS) state. The CN5 HS complex is further stabilized by the coordination of a sixth ligand, resulting in minor changes of the spectroscopic properties of the CN6 complexes compared to their CN5 counterparts. The coordination and de-coord­in­ation of axial ligands are observed in a fast dynamic equilibrium, dominated by the CN4 and the CN6 species (Kadish *et al.*, 2000 ▸ and Kruglik *et al.*, 2003 ▸). The spectra and properties of a well defined five-coordinate (CN5) Ni^II^ porphyrin in solution and the solid state was described recently (Gutzeit *et al.*, 2019*a* ▸). In closely related, tightly strapped Ni^II^ porphyrins, the coordination of the axial pyridine ligand is dependent on the geometry of the ligand-containing strap (Köbke *et al.*, 2019 ▸). Furthermore, the coordination behaviour is dependent on the *para* substituent of the pyridine moiety due to its electronic influence (Dommaschk *et al.*, 2014 ▸). Hence, a *para* methyl substituent was introduced in the complex described previously (Gutzeit *et al.*, 2019*a* ▸) to improve the intra­molecular coordination. The modified synthesis yielded the title compound as a byproduct (Gutzeit *et al.*, 2019*a* ▸; Köbke *et al.*, 2019 ▸) similar to the synthesis of the unsubstituted derivative (Gutzeit *et al.*, 2019*b* ▸). Metallation was achieved under standard conditions. Splitting of the CH_2_-proton signals in the ^1^H NMR spectrum are observed for the unmetallated porphyrin and the title compound due to an impeded ring inversion of the strap (Gutzeit *et al.*, 2019*b* ▸). The increased paramagnetic shifts of the β-pyrrole H atoms (δ_min_ = 8.8 ppm, δ_max_ = 49.0 ppm, CDCl_3_, 298 K; Gutzeit *et al.*, 2019*a* ▸) of the title compound (45.9 ppm) compared to the compound without a methyl group in *para* position of the pyridine ring (42.2 ppm) indicates an increase of intra­molecular coordination by 9% **(**Fig. 1 ▸; Gutzeit *et al.*, 2019*a* ▸), confirming the influence of the *para* methyl substituent.
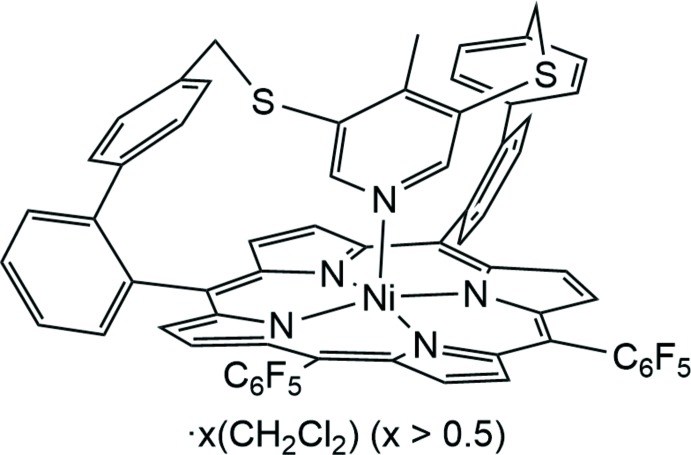


## Structural commentary   

**Figure 2:**
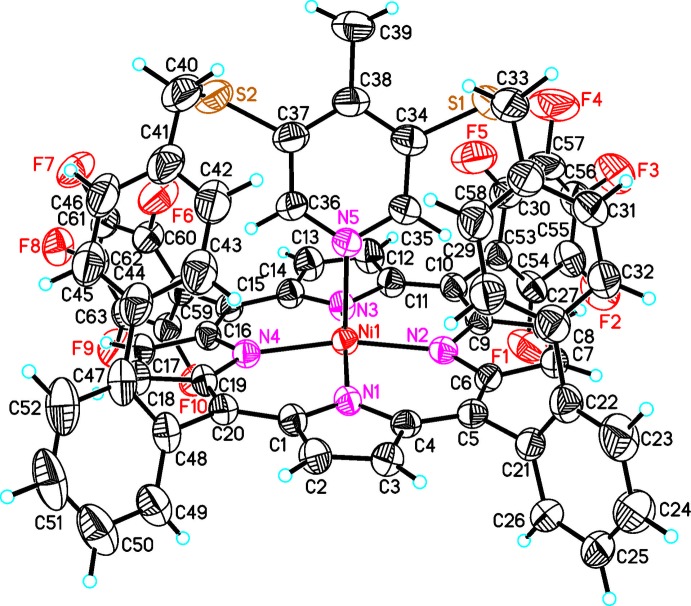
The mol­ecular structure of the title compound with displacement ellipsoids drawn at the 50% probability level.

**Figure 3:**
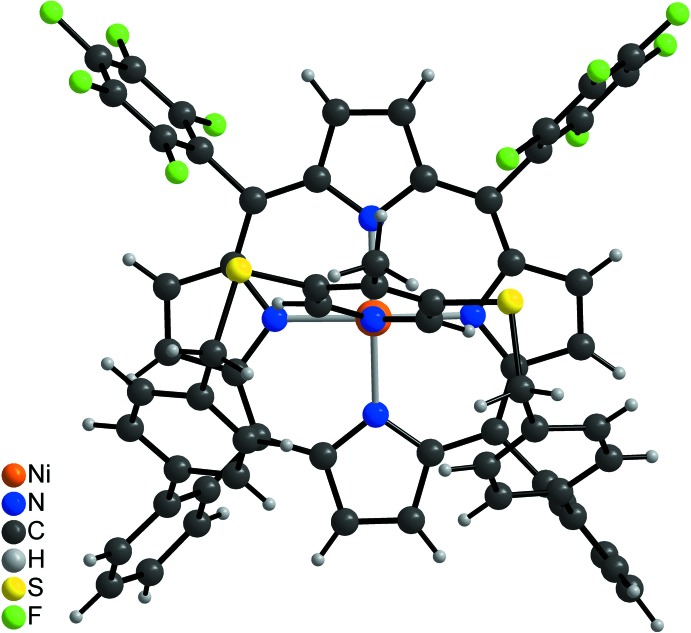
Mol­ecular structure of the title compound viewed onto the porphyrin plane.

**Figure 5:**
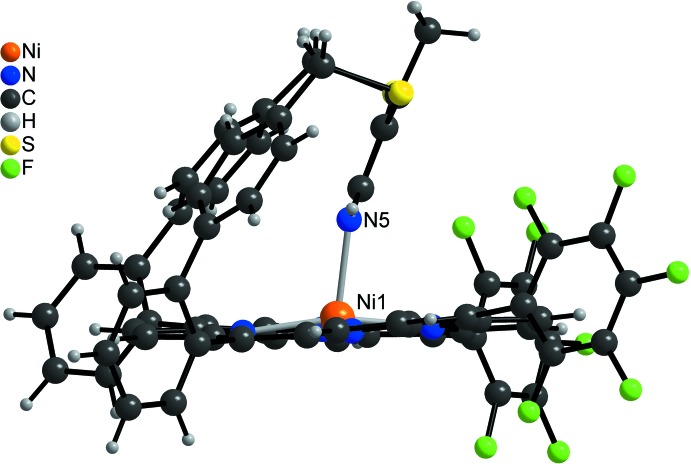
Mol­ecular structure of the title compound showing the orientation of the pyridine ring relative to the N_4_ plane.

In the crystal structure of the title compound, (C_64_H_33_F_10_N_5_NiS_2_) (CH_2_Cl_2_)_x_, the five-coordinate Ni^II^ cations are bound by the four nitro­gen atoms of the porphyrin mol­ecule and the nitro­gen atom of the pyridine ring (Figs. 2 ▸–4 ▸ ▸). The porphyrin plane is distorted due to steric constraints of the strap, similar to the unsubstituted derivative (Gutzeit *et al.*, 2019*b* ▸). The maximum deviation of the individual atoms from the mean plane calculated through the porphyrin atoms amounting to 0.137 (3) Å for the parent compound (Gutzeit *et al.*, 2019*b* ▸) is increased to 0.159 (4) Å in the title compound. The Ni—N bond lengths to the porphyrin nitro­gen atoms [2.031 (3)–2.041 (3) Å] are significantly shorter than that to the pyridine nitro­gen atom (Table 1 ▸). In the title compound, the Ni^II^ cation is shifted 0.241 (3) Å out of the porphyrin N4 plane towards the pyridine nitro­gen atom, which is slightly shorter than that in the derivative without the methyl group [0.250 (3) Å, Fig. 5 ▸]. This is also the case for the Ni—N distance to the pyridine N atom of 2.106 (3) Å, compared to 2.112 (2) Å in the derivative. The angle between the planes of the pyridine ring and the N_4_ porphyrin plane amounts to 67.1 (2)°, which is very different from that in the derivative without the methyl group [80.48 (6)°; Fig. 5 ▸]. The tilt of the pyridine ring does not impede the intra­molecular coordination, which is reflected by the short Ni—N_py_ (py = pyridine) distance and the NMR shift. The tilt of the axial ligand is reinforced by packing effects leveraged by the *para* methyl group. This is also in agreement with a different conformation of the overall porphyrin mol­ecule compared to the unsubstituted derivative, because the penta­fluoro phenyl rings are more perpendicular to the porphyrin N4 plane with dihedral angles of 82.53 (8) and 77.37 (7)°, which is also the case for the phenyl rings [67.0 (1) and 83.4 (2)°; Figs. 3 ▸ and 4 ▸]. Finally, the dihedral angles between the biphenyl rings are 72.3 (2) and 64.3 (2) ° compared to 63.2 (1) and 53.5 (1)° in the derivative. Overall, the increased steric demand of the *para* methyl substituent increases the distortion compared to the unsubstituted derivative.

## Supra­molecular features   

**Figure 6:**
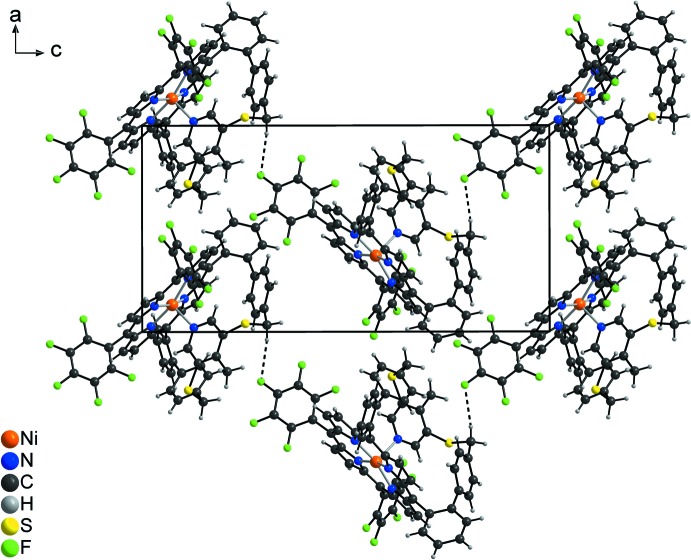
Crystal structure of the title compound viewed down [010] with inter­molecular C—H⋯F hydrogen bonds shown as dashed lines.

In the extended structure of the title compound, the complexes are linked by C—H⋯F hydrogen bonds into zigzag chains that extend in the [001] direction with adjacent complexes related by a 2_1_-screw-axis (Fig. 6 ▸). The C—H⋯F angle is 164°, indicating a relative strong inter­action (Table 2 ▸). By this arrangement, cavities are formed, in which the disordered di­chloro­methane solvate mol­ecules are located. There are additional intra­molecular C—H⋯N contacts, with angles far from linearity that correspond to only very weak inter­actions (Table 2 ▸).

## Database survey   

According to a search of the Cambridge Structural Database, only four crystal structures of five-coordinate Ni^II^ porphyrins have been reported (Kumar & Sankar, 2014 ▸; Dommaschk *et al.*, 2015*c* ▸; Gutzeit *et al.*, 2019*a* ▸,*b* ▸; refcodes DOJPAV01, QUZVAK, COCBAA and HOPSIR, respectively). The square-pyramidal complex geometry is predominant in zinc (Paul *et al.*, 2003 ▸; Deutman *et al.*, 2014 ▸) and iron (Awasabisah *et al.*, 2015 ▸; Yu *et al.*, 2015 ▸) porphyrins. Zinc porphyrins form five-coordinate complexes additionally with oxygen-containing ligands (Leben *et al.*, 2018 ▸), a behaviour uncommon in Ni^II^ porphyrins (Ozette *et al.*, 1997 ▸). The conformation of the porphyrin (Flanagan *et al.*, 2015 ▸; Senge, 2011 ▸) has been recognized as an important factor for the axial coordination, spin state (Thies *et al.*, 2010 ▸; Dommaschk *et al.*, 2014 ▸) and catalytic activity (Ramesh *et al.*, 2016 ▸) of these complexes.

## Synthesis and crystallization   

The free base porphyrin of the title compound was obtained as a byproduct of a variant of the published procedure (Gutzeit *et al.*, 2019*a* ▸; Köbke *et al.*, 2019 ▸). The free base porphyrins were separated by column chromatography (silica gel, di­chloro­methane; silica gel, di­chloro­methane/n-pentane, 1:1 and silica gel, toluene) and precipitated from di­chloro­methane by diffusion of methanol (59 mg, 3%).

^1^H NMR (500 MHz, CDCl_3_, 298 K, TMS): δ = 8.97 (*s*, 2 H, *H*_β,Por_), 8.65–8.58 (*m*, 4 H, *H*_β,Por_), 8.51 (*d*, ^3^ *J* = 4.8 Hz, 2 H, *H*_β,Por_), 8.26 (*dd*, ^3^ *J* = 7.5 Hz, ^4^ *J* = 1.1 Hz, 2 H, *H*-3_BP_), 7.91 (*td*, ^3^ *J* = 7.7 Hz, ^4^ *J* = 1.4 Hz, 2 H, *H*-5_BP_), 7.83 (*dd*, ^3^ *J* = 7.9 Hz, ^4^ *J* = 1.1 Hz, 2 H, *H*-6_BP_), 7.75 (*td*, ^3^ *J* = 7.5 Hz, ^4^ *J* = 1.4 Hz, 2 H, *H*-4_BP_), 6.66 (*d*, ^3^ *J* = 8.2 Hz, 4 H, *H*-2′_BP_), 5.67 (*d*, ^3^ *J* = 8.2 Hz, 4 H, *H*-3′_BP_), 3.00–2.90 (*m*, 4 H, C*H*_2,a+b_), 2.21 (*s*, 3 H, C*H*_3_), −2.82 (*s*, 2 H, N*H*) ppm. Unobserved signals: *H*-2_Py_. ^13^C NMR (126 MHz, CDCl_3_, 298 K): δ = 153.5 (*C*4_Py_), 144.5 (*C*1_BP_), 140.2 (*C*1′_BP_), 139.9 (*C*2_BP_), 135.9 (*C*4′_BP_), 134.7 (*C*3_BP_), 129.9 (*C*3_Py_), 129.4 (*C*6_BP_), 129.3 (*C*2′_BP_), 129.2 (*C*5_BP_), 127.4 (*C*3′_BP_), 125.8 (*C*4_BP_), 121.4 (*C*5Por,*C*10Por), 38.3 (*C*H_2_), 17.6 (*C*H_3_) ppm. Unobserved signals: *C*15_Por_, *C*20_Por_, *C*_α,Por_, *C*_β,Por_, *C*_6_F_5_. ^19^F NMR (471 MHz, CDCl_3_, 298 K): δ = −136.96 (*dd*, ^3^ *J* = 24.3 Hz, ^4^ *J* = 8.3 Hz, *F*-*ortho*), −137.26 (*dd*, ^3^ *J* = 24.7 Hz, ^4^ *J* = 8.1 Hz, *F*-*ortho*), −153.08 (t, ^3^ *J* = 20.0 Hz, *F*-*para*), −162.43 to −162.65 (*m*, *F*-*meta*) ppm. FT–IR (ATR): ν = 2342.6 (*w*), 2326.3 (*w*), 1742.5 (*w*), 1516.5 (*s*), 1493.9 (*s*), 1474.0 (*s*), 1422.1 (*w*), 1348.9 (*w*), 1217.9 (*w*), 1078.8 (*w*), 1041.7 (*w*), 985.4 (*s*), 917.9 (*s*), 839.4 (*w*), 800.8 (*s*), 763.4 (*s*), 737.1 (*s*), 716.5 (*s*), 700.0 (*m*), 659.5 (*m*), 526.8 (*w*), 506.0 (*w*), 467.0 (*w*), 407.2 (*m*) cm^−1^. MS (EI): *m*/*z* (%) = 1188.10 (43) [*M* − H_2_ + Cu]^+^, 334.96 (14) [*M* - C_44_H_18_F_10_N_4_]^+^, 168.99 (100) [*M* − C_57_H_28_F_10_N_4_]^+^ u. HRMS (EI): Calculated for C_64_H_33_CuF_10_N_5_S_2_: 1188.1314 u. Found: 1188.128 33 u. Diff.: 2.6 ppm. The free base porphyrin is metallated in the process of sublimation. EA: Calculated for C_64_H_35_F_10_N_5_S_2_·0.5(CH_2_Cl_2_): C 66.18, H 3.10, N 5.98, S 5.48. Found C 66.77, H 3.39, N 5.44, S 5.33.

The nickel cation was introduced under standard conditions (31 mg porphyrin, 68 mg Ni(acac)_2_, 30 ml toluene, reflux, 21 h) followed by filtration through a pluck of silica (di­chloro­methane) and precipitation from di­chloro­methane by diffusion of methanol. The crystals were washed with methanol and *n*-pentane (11 mg, 34%).

^1^H NMR (500 MHz, CDCl_3_, 298 K, TMS, TFA): δ = 9.00–8.32 (*m*, 8 H, *H*_β,Por_), 7.97 (*dd*, ^3^ *J* = 7.7 Hz, ^4^ *J* = 1.3 Hz, 2 H, *H*-3_BP_), 7.87 (*td*, ^3^ *J* = 7.7 Hz, ^4^ *J* = 1.1 Hz, 2 H, *H*-5_BP_), 7.79 (*dd*, ^3^ *J* = 7.8 Hz, ^4^ *J* = 1.1 Hz, 2 H, *H*-6_BP_), 7.69 (*td*, ^3^ *J* = 7.8 Hz, ^4^ *J* = 1.3 Hz, 2 H, *H*-4_BP_), 6.76 (*d*, ^3^ *J* = 8.1 Hz, 4 H, *H*-2′_BP_), 6.56 (*s*, 2 H, *H*-2_Py_), 6.08 (*d*, ^3^ *J* = 8.1 Hz, 4 H, *H*-3′_BP_), 3.54-3.42 (*m*, 4 H, C*H*_2,a+b_), 2.38 (*s*, 3 H, C*H*_3_) ppm. ^13^C NMR (126 MHz, CDCl_3_, 298 K, TFA): δ = 165.0 (*C*4_Py_), 143.0 (*C*1_BP_), 141.5 (*C*1′_BP_), 138.9 (*C*2_Py_), 137.3 (*C*3_Py_), 135.0 (*C*3_BP_), 133.3 (*C*4′_BP_), 129.9 (*C*6_BP_), 129.7 (*C*5_BP_), 129.6 (*C*2′_BP_), 127.8 (*C*3′_BP_), 126.6 (*C*4_BP_), 37.8 (*C*H_2_), 19.3 (*C*H_3_) ppm. Unobserved signals: *C_meso_*_,Por_, *C*_α,Por_, *C*_β_,Por, *C*_6_F_5_. ^19^F NMR (471 MHz, CDCl_3_, 298 K, TFA): δ = −137.27 (*br*, *F*-*ortho*), −138.66 (*br*, *F*-*ortho*), −152.09 (*t*, ^3^ *J* = 20.5 Hz, *F*-*para*), −161.65 (*td*, ^3^ *J* = 22.0 Hz, ^4^ *J* = 8.2 Hz, *F*-*meta*), −162.06 (*td*, ^3^ *J* = 22.0 Hz, ^4^ *J* = 8.3 Hz, *F*-*meta*) ppm. FT–IR (ATR): ν = 1517.8 (*m*), 1487.0 (*m*), 1338.8 (*w*), 1065.1 (*w*), 986.5 (*s*), 948.7 (*m*), 928.9 (*s*), 835.3 (*w*), 799.5 (*m*), 766.4 (*m*), 752.8 (*m*), 707.9 (*w*), 664.1 (*w*), 599.9 (*w*), 535.7 (*w*), 431.5 (*w*), 418.6 (*w*) cm^−1^. MS (EI): m/z (%) = 1183.10 (32) [*M*]^+^, 169.00 (86) [*M* − C_57_H_26_F_10_N_4_Ni]^+^, 131.00 (100) [*M* − C_57_H_33_F_10_N_4_NiS]^+^ u. HRMS (EI): Calculated for C_64_H_33_F_10_N_5_NiS_2_: 1183.1371 u. Found: 1183.1362 u. Diff.: 0.8 ppm.

Red blocks of the title compound were obtained by dissolving the complex in di­chloro­methane and gas-phase diffusion of methanol.

## Refinement   

Crystal data, data collection and structure refinement details are summarized in Table 3 ▸. The C—H hydrogen atoms were located in difference maps but were positioned with idealized geometry (C—H = 0.95–0.98 Å) and refined isotropically with *U*_iso_(H) = 1.2*U*_eq_(C) or 1.5*U*_eq_(C-meth­yl) using a riding model.

After structure refinement using a model with one Ni porphyrin complex and a half di­chloro­methane solvate mol­ecule disordered around a center of inversion, there was significant residual electron density that definitely corres­ponds to additional di­chloro­methane disordered over several orientations. A number of different split models were tried using restraints for the geometry and for the components of the anisotropic displacement parameters, but no reasonable structural model was found and very large anisotropic displacement parameters were obtained. Therefore, the contribution of this solvent to the electron density was removed with SQUEEZE in *PLATON* (Spek, 2009 ▸, 2015 ▸), which leads to a reasonable structure model and very good reliability factors. By this procedure, the amount of di­chloro­methane cannot accurately be determined and there is indication that this position is not fully occupied, which is highly likely because this solvate is very unstable and already starts to decompose during the sample preparation.

## Supplementary Material

Crystal structure: contains datablock(s) I. DOI: 10.1107/S2056989019012453/hb7846sup1.cif

Structure factors: contains datablock(s) I. DOI: 10.1107/S2056989019012453/hb7846Isup2.hkl

CCDC reference: 1951945

Additional supporting information:  crystallographic information; 3D view; checkCIF report

## Figures and Tables

**Figure 4:**
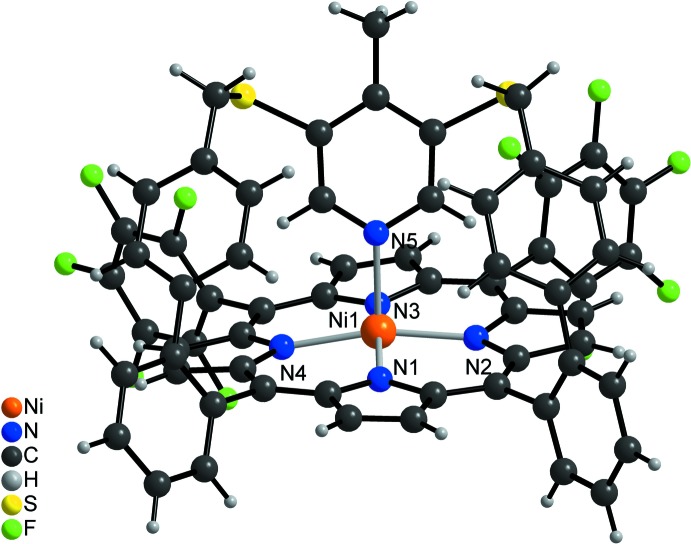
Mol­ecular structure of the title compound showing the square-pyramidal Ni^II^ coordination.

**Table 1:** Selected bond lengths (Å)

Ni1—N2	2.031 (3)	Ni1—N1	2.041 (3)
Ni1—N4	2.036 (3)	Ni1—N5	2.106 (3)
Ni1—N3	2.036 (3)		

**Table 2:** Hydrogen-bond geometry (Å, °)

*D*—H⋯*A*	*D*—H	H⋯*A*	*D*⋯*A*	*D*—H⋯*A*
C33—H33*B*⋯F8^i^	0.99	2.63	3.592 (6)	164
C35—H35⋯N2	0.95	2.58	3.125 (5)	117
C36—H36⋯N4	0.95	2.60	3.206 (5)	122

Symmetry code: (i) 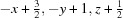.

**Table 3:** Experimental details

Crystal data	
Chemical formula	[Ni(C_64_H_33_F_10_N_5_S_2_)][+solvent]
*M*_r_	1184.78
Crystal system, space group	Orthorhombic, *P*2_1_2_1_2_1_
Temperature (K)	170
*a*, *b*, *c* (Å)	12.6269 (2), 18.0525 (3), 24.9524 (6)
*V* (Å^3^)	5687.83 (19)
*Z*	4
Radiation type	Mo *K*α
μ (mm^−1^)	0.49
Crystal size (mm)	0.15 × 0.10 × 0.05

Data collection	
Diffractometer	Stoe *IPDS2*
Absorption correction	Numerical (*X-RED* and *X-SHAPE*; Stoe, 2008 ▸)
*T*_min_, *T*_max_	0.810, 0.965
No. of measured, independent and observed [*I* > 2σ(*I*)] reflections	44401, 12417, 10468
*R*_int_	0.055
(sin θ/λ)_max_ (Å^−1^)	0.639

Refinement	
*R*[*F* ^2^ > 2σ(*F* ^2^)], *wR*(*F* ^2^), *S*	0.043, 0.099, 1.04
No. of reflections	12417
No. of parameters	740
H-atom treatment	H-atom parameters constrained
Δρ_max_, Δρ_min_ (e Å^−3^)	0.34, −0.39
Absolute structure	Flack *x* determined using 4043 quotients [(*I* ^+^)−(*I* ^−^)]/[(*I* ^+^)+(*I* ^−^)] (Parsons et al., 2013 ▸)
Absolute structure parameter	0.004 (7)

Computer programs: *X-AREA* (Stoe, 2008 ▸), *SHELXT* (Sheldrick, 2015*a* ▸), *SHELXL2014* (Sheldrick, 2015*b* ▸), *XP* (Sheldrick, 2008 ▸), *DIAMOND* (Brandenburg, 2014 ▸) and *publCIF* (Westrip, 2010 ▸).

## supplementary crystallographic information

### Crystal data

**Table:**

[Ni(C_64_H_33_F_10_N_5_S_2_)][+solvent]	*D*_x_ = 1.384 Mg m^−^^3^
*M**_r_* = 1184.78	Mo *K*α radiation, λ = 0.71073 Å
Orthorhombic, *P*2_1_2_1_2_1_	Cell parameters from 44396 reflections
*a* = 12.6269 (2) Å	θ = 1.4–27.0°
*b* = 18.0525 (3) Å	µ = 0.49 mm^−^^1^
*c* = 24.9524 (6) Å	*T* = 170 K
*V* = 5687.83 (19) Å^3^	Block, red
*Z* = 4	0.15 × 0.10 × 0.05 mm
*F*(000) = 2408	

### Data collection

**Table:**

Stoe IPDS-2 diffractometer	10468 reflections with *I* > 2σ(*I*)
ω scans	*R*_int_ = 0.055
Absorption correction: numerical (X-Red and X-Shape; Stoe, 2008)	θ_max_ = 27.0°, θ_min_ = 1.4°
*T*_min_ = 0.810, *T*_max_ = 0.965	*h* = −16→14
44401 measured reflections	*k* = −23→20
12417 independent reflections	*l* = −31→31

### Refinement

**Table:**

Refinement on *F*^2^	Hydrogen site location: inferred from neighbouring sites
Least-squares matrix: full	H-atom parameters constrained
*R*[*F*^2^ > 2σ(*F*^2^)] = 0.043	*w* = 1/[σ^2^(*F*_o_^2^) + (0.0479*P*)^2^ + 0.9016*P*] where *P* = (*F*_o_^2^ + 2*F*_c_^2^)/3
*wR*(*F*^2^) = 0.099	(Δ/σ)_max_ < 0.001
*S* = 1.04	Δρ_max_ = 0.34 e Å^−^^3^
12417 reflections	Δρ_min_ = −0.39 e Å^−^^3^
740 parameters	Absolute structure: Flack *x* determined using 4043 quotients [(*I*^+^)-(*I*^-^)]/[(*I*^+^)+(*I*^-^)] (Parsons et al., 2013)
0 restraints	Absolute structure parameter: 0.004 (7)

### Special details

**Table:**

Geometry. All esds (except the esd in the dihedral angle between two l.s. planes) are estimated using the full covariance matrix. The cell esds are taken into account individually in the estimation of esds in distances, angles and torsion angles; correlations between esds in cell parameters are only used when they are defined by crystal symmetry. An approximate (isotropic) treatment of cell esds is used for estimating esds involving l.s. planes.

### Fractional atomic coordinates and isotropic or equivalent isotropic displacement parameters (Å^2^)

**Table:**

	*x*	*y*	*z*	*U*_iso_*/*U*_eq_	
Ni1	0.36866 (4)	0.61346 (3)	0.57341 (2)	0.03235 (11)	
N1	0.3385 (2)	0.71635 (17)	0.60371 (11)	0.0329 (7)	
N2	0.2384 (3)	0.57504 (17)	0.61227 (12)	0.0340 (7)	
N3	0.3742 (3)	0.51813 (17)	0.52990 (11)	0.0374 (7)	
N4	0.4729 (3)	0.66068 (18)	0.52138 (11)	0.0337 (7)	
C1	0.3978 (3)	0.7789 (2)	0.59612 (14)	0.0361 (8)	
C2	0.3622 (4)	0.8368 (2)	0.63062 (15)	0.0411 (8)	
H2	0.3916	0.8851	0.6336	0.049*	
C3	0.2787 (3)	0.8098 (2)	0.65832 (15)	0.0385 (8)	
H3	0.2376	0.8358	0.6841	0.046*	
C4	0.2636 (3)	0.7342 (2)	0.64145 (14)	0.0335 (8)	
C5	0.1883 (3)	0.6867 (2)	0.66275 (14)	0.0348 (8)	
C6	0.1780 (3)	0.6117 (2)	0.64944 (13)	0.0342 (7)	
C7	0.1072 (3)	0.5608 (2)	0.67541 (15)	0.0387 (9)	
H7	0.0574	0.5724	0.7027	0.046*	
C8	0.1249 (4)	0.4939 (2)	0.65372 (15)	0.0429 (9)	
H8	0.0898	0.4491	0.6629	0.052*	
C9	0.2067 (3)	0.5023 (2)	0.61405 (15)	0.0378 (8)	
C10	0.2491 (3)	0.4457 (2)	0.58317 (14)	0.0382 (8)	
C11	0.3253 (3)	0.4528 (2)	0.54297 (15)	0.0388 (8)	
C12	0.3605 (4)	0.3948 (2)	0.50759 (17)	0.0496 (10)	
H12	0.3392	0.3444	0.5084	0.060*	
C13	0.4297 (4)	0.4262 (3)	0.47302 (17)	0.0497 (10)	
H13	0.4650	0.4019	0.4443	0.060*	
C14	0.4400 (3)	0.5026 (2)	0.48752 (15)	0.0385 (8)	
C15	0.5083 (3)	0.5531 (2)	0.46350 (14)	0.0382 (8)	
C16	0.5222 (3)	0.6268 (2)	0.47900 (13)	0.0342 (8)	
C17	0.5939 (3)	0.6784 (2)	0.45336 (16)	0.0423 (9)	
H17	0.6376	0.6687	0.4232	0.051*	
C18	0.5869 (3)	0.7423 (2)	0.48022 (15)	0.0417 (9)	
H18	0.6247	0.7865	0.4726	0.050*	
C19	0.5114 (3)	0.7316 (2)	0.52279 (14)	0.0359 (8)	
C20	0.4805 (3)	0.7864 (2)	0.55899 (14)	0.0368 (8)	
C21	0.1169 (3)	0.7200 (2)	0.70420 (15)	0.0385 (8)	
C22	0.1413 (4)	0.7145 (3)	0.75868 (16)	0.0480 (10)	
C23	0.0784 (5)	0.7530 (3)	0.7952 (2)	0.0701 (16)	
H23	0.0942	0.7501	0.8324	0.084*	
C24	−0.0060 (5)	0.7952 (3)	0.7784 (2)	0.0754 (17)	
H24	−0.0465	0.8221	0.8039	0.090*	
C25	−0.0319 (5)	0.7984 (3)	0.7248 (2)	0.0630 (14)	
H25	−0.0918	0.8261	0.7134	0.076*	
C26	0.0293 (4)	0.7613 (3)	0.68774 (18)	0.0481 (10)	
H26	0.0118	0.7639	0.6508	0.058*	
C27	0.2296 (4)	0.6651 (3)	0.77658 (16)	0.0495 (11)	
C28	0.3349 (4)	0.6799 (3)	0.76606 (18)	0.0562 (12)	
H28	0.3544	0.7260	0.7506	0.067*	
C29	0.4124 (4)	0.6277 (3)	0.77794 (18)	0.0589 (13)	
H29	0.4847	0.6393	0.7716	0.071*	
C30	0.3863 (4)	0.5589 (3)	0.79893 (17)	0.0572 (12)	
C31	0.2806 (4)	0.5464 (3)	0.8117 (2)	0.0631 (14)	
H31	0.2611	0.5011	0.8284	0.076*	
C32	0.2040 (4)	0.5979 (3)	0.80093 (18)	0.0584 (13)	
H32	0.1324	0.5878	0.8101	0.070*	
C33	0.4666 (4)	0.4992 (4)	0.80592 (18)	0.0669 (15)	
H33A	0.4555	0.4748	0.8410	0.080*	
H33B	0.5382	0.5215	0.8061	0.080*	
S1	0.45960 (12)	0.42922 (8)	0.75272 (5)	0.0652 (4)	
C34	0.5137 (4)	0.4798 (3)	0.69782 (17)	0.0488 (10)	
C35	0.4473 (4)	0.5274 (2)	0.67001 (16)	0.0442 (10)	
H35	0.3763	0.5329	0.6820	0.053*	
N5	0.4788 (3)	0.5663 (2)	0.62680 (12)	0.0397 (7)	
C36	0.5793 (3)	0.5587 (3)	0.61168 (17)	0.0449 (9)	
H36	0.6028	0.5858	0.5813	0.054*	
C37	0.6515 (4)	0.5137 (3)	0.63743 (17)	0.0516 (11)	
C38	0.6190 (4)	0.4710 (3)	0.68145 (17)	0.0494 (10)	
C39	0.6924 (5)	0.4168 (3)	0.7083 (2)	0.0659 (14)	
H39A	0.6722	0.4113	0.7460	0.099*	
H39B	0.7653	0.4351	0.7060	0.099*	
H39C	0.6873	0.3687	0.6903	0.099*	
S2	0.78309 (11)	0.51140 (10)	0.61283 (6)	0.0702 (4)	
C40	0.8384 (4)	0.5904 (4)	0.6494 (2)	0.080 (2)	
H40A	0.8259	0.5830	0.6882	0.095*	
H40B	0.9159	0.5917	0.6436	0.095*	
C41	0.7924 (4)	0.6637 (4)	0.63313 (19)	0.0633 (14)	
C42	0.7113 (4)	0.6972 (4)	0.66102 (18)	0.0649 (15)	
H42	0.6871	0.6754	0.6935	0.078*	
C43	0.6643 (4)	0.7615 (3)	0.64298 (18)	0.0607 (13)	
H43	0.6077	0.7828	0.6627	0.073*	
C44	0.6989 (4)	0.7953 (3)	0.59624 (17)	0.0518 (11)	
C45	0.7836 (4)	0.7635 (3)	0.5695 (2)	0.0630 (13)	
H45	0.8105	0.7867	0.5382	0.076*	
C46	0.8301 (4)	0.6984 (4)	0.5873 (2)	0.0658 (14)	
H46	0.8878	0.6775	0.5681	0.079*	
C47	0.6447 (4)	0.8620 (3)	0.57452 (17)	0.0516 (10)	
C48	0.5386 (4)	0.8582 (2)	0.55788 (16)	0.0442 (10)	
C49	0.4884 (5)	0.9215 (3)	0.53857 (18)	0.0548 (12)	
H49	0.4171	0.9190	0.5264	0.066*	
C50	0.5429 (6)	0.9883 (3)	0.5372 (2)	0.0734 (17)	
H50	0.5077	1.0321	0.5259	0.088*	
C51	0.6486 (6)	0.9911 (3)	0.5521 (2)	0.0768 (19)	
H51	0.6863	1.0366	0.5497	0.092*	
C52	0.6982 (5)	0.9296 (3)	0.5701 (2)	0.0661 (15)	
H52	0.7707	0.9323	0.5800	0.079*	
C53	0.2096 (4)	0.3686 (2)	0.59367 (16)	0.0428 (9)	
C54	0.1119 (4)	0.3441 (2)	0.57611 (18)	0.0492 (10)	
C55	0.0757 (4)	0.2733 (3)	0.58575 (19)	0.0549 (12)	
C56	0.1364 (5)	0.2256 (2)	0.6139 (2)	0.0609 (12)	
C57	0.2319 (5)	0.2470 (3)	0.6320 (2)	0.0666 (14)	
C58	0.2688 (4)	0.3177 (3)	0.6224 (2)	0.0569 (12)	
F1	0.0481 (3)	0.38982 (19)	0.54925 (13)	0.0742 (9)	
F2	−0.0200 (3)	0.25128 (18)	0.56817 (14)	0.0788 (9)	
F3	0.0995 (3)	0.15736 (17)	0.62517 (16)	0.0862 (11)	
F4	0.2921 (4)	0.2008 (2)	0.6617 (2)	0.1099 (15)	
F5	0.3640 (3)	0.33798 (18)	0.64045 (15)	0.0827 (10)	
C59	0.5742 (3)	0.5266 (2)	0.41772 (15)	0.0400 (9)	
C60	0.6774 (3)	0.5037 (3)	0.42437 (17)	0.0475 (9)	
C61	0.7405 (4)	0.4800 (3)	0.38280 (17)	0.0472 (10)	
C62	0.6994 (4)	0.4791 (2)	0.33204 (16)	0.0459 (10)	
C63	0.5979 (4)	0.5029 (3)	0.32304 (15)	0.0456 (10)	
C64	0.5368 (3)	0.5260 (3)	0.36550 (16)	0.0437 (9)	
F6	0.7198 (2)	0.5046 (2)	0.47407 (10)	0.0686 (8)	
F7	0.8392 (2)	0.4563 (2)	0.39158 (12)	0.0704 (9)	
F8	0.7586 (2)	0.45350 (17)	0.29130 (10)	0.0613 (7)	
F9	0.5582 (3)	0.50154 (19)	0.27319 (10)	0.0686 (8)	
F10	0.4375 (2)	0.54870 (18)	0.35559 (10)	0.0598 (7)	

### Atomic displacement parameters (Å^2^)

**Table:**

	*U*^11^	*U*^22^	*U*^33^	*U*^12^	*U*^13^	*U*^23^
Ni1	0.0320 (2)	0.0340 (2)	0.03107 (19)	−0.0007 (2)	0.00063 (19)	0.00143 (18)
N1	0.0336 (17)	0.0328 (16)	0.0324 (15)	−0.0034 (13)	0.0006 (12)	0.0022 (12)
N2	0.0353 (17)	0.0312 (16)	0.0354 (14)	0.0007 (14)	−0.0009 (13)	0.0008 (12)
N3	0.0395 (17)	0.0364 (16)	0.0364 (14)	−0.0033 (16)	0.0048 (14)	0.0013 (12)
N4	0.0329 (16)	0.0352 (17)	0.0330 (14)	−0.0004 (14)	0.0005 (12)	0.0007 (12)
C1	0.037 (2)	0.0354 (19)	0.0363 (16)	−0.0058 (16)	0.0008 (14)	0.0015 (15)
C2	0.042 (2)	0.037 (2)	0.0441 (19)	−0.0073 (19)	−0.0005 (18)	−0.0016 (15)
C3	0.039 (2)	0.038 (2)	0.0377 (18)	−0.0006 (18)	0.0012 (16)	−0.0038 (15)
C4	0.0333 (19)	0.0346 (19)	0.0324 (16)	0.0029 (16)	−0.0022 (14)	0.0011 (14)
C5	0.033 (2)	0.038 (2)	0.0335 (17)	−0.0002 (16)	−0.0038 (14)	0.0002 (15)
C6	0.0290 (16)	0.0396 (19)	0.0339 (16)	0.0009 (18)	0.0006 (13)	0.0016 (16)
C7	0.037 (2)	0.042 (2)	0.0370 (17)	−0.0016 (17)	0.0042 (15)	0.0023 (16)
C8	0.044 (2)	0.037 (2)	0.0482 (19)	−0.006 (2)	0.0050 (18)	0.0063 (16)
C9	0.038 (2)	0.035 (2)	0.0404 (18)	−0.0072 (17)	−0.0003 (16)	0.0038 (16)
C10	0.041 (2)	0.0336 (19)	0.0398 (19)	−0.0007 (17)	0.0039 (16)	0.0031 (15)
C11	0.043 (2)	0.0299 (19)	0.0438 (19)	−0.0038 (17)	0.0031 (16)	−0.0017 (16)
C12	0.059 (3)	0.036 (2)	0.054 (2)	−0.005 (2)	0.011 (2)	−0.0063 (18)
C13	0.057 (3)	0.045 (2)	0.047 (2)	−0.001 (2)	0.011 (2)	−0.0063 (19)
C14	0.040 (2)	0.037 (2)	0.0382 (18)	0.0011 (18)	0.0052 (15)	−0.0037 (16)
C15	0.037 (2)	0.044 (2)	0.0340 (17)	0.0013 (18)	0.0035 (15)	−0.0002 (16)
C16	0.0361 (19)	0.039 (2)	0.0275 (15)	−0.0013 (16)	0.0032 (13)	0.0017 (14)
C17	0.042 (2)	0.048 (2)	0.0376 (19)	−0.0022 (19)	0.0075 (16)	0.0015 (17)
C18	0.040 (2)	0.044 (2)	0.0409 (19)	−0.0069 (18)	0.0082 (16)	0.0017 (17)
C19	0.036 (2)	0.037 (2)	0.0341 (17)	−0.0068 (17)	0.0034 (15)	0.0038 (15)
C20	0.036 (2)	0.036 (2)	0.0382 (18)	−0.0024 (16)	0.0012 (15)	0.0018 (15)
C21	0.036 (2)	0.036 (2)	0.0427 (18)	−0.0001 (17)	0.0065 (16)	−0.0001 (15)
C22	0.051 (3)	0.052 (2)	0.0406 (19)	0.000 (2)	0.0085 (19)	−0.0008 (17)
C23	0.094 (4)	0.073 (4)	0.043 (2)	0.013 (3)	0.024 (3)	−0.003 (2)
C24	0.093 (4)	0.070 (4)	0.064 (3)	0.030 (3)	0.031 (3)	−0.001 (3)
C25	0.063 (3)	0.054 (3)	0.073 (3)	0.020 (3)	0.032 (3)	0.011 (2)
C26	0.043 (2)	0.049 (3)	0.052 (2)	0.008 (2)	0.0078 (19)	0.0059 (19)
C27	0.051 (3)	0.062 (3)	0.0351 (19)	0.000 (2)	0.0004 (18)	0.0011 (18)
C28	0.051 (3)	0.069 (3)	0.049 (2)	−0.007 (2)	0.000 (2)	0.004 (2)
C29	0.047 (3)	0.085 (4)	0.045 (2)	−0.006 (3)	−0.0011 (19)	0.006 (2)
C30	0.048 (3)	0.084 (4)	0.039 (2)	0.004 (3)	−0.0033 (19)	0.011 (2)
C31	0.052 (3)	0.083 (4)	0.054 (3)	0.002 (3)	0.001 (2)	0.027 (3)
C32	0.045 (2)	0.080 (4)	0.050 (2)	−0.003 (3)	0.0022 (19)	0.020 (2)
C33	0.053 (3)	0.106 (4)	0.042 (2)	0.014 (3)	−0.003 (2)	0.021 (3)
S1	0.0664 (8)	0.0700 (9)	0.0591 (7)	0.0039 (7)	−0.0009 (6)	0.0279 (6)
C34	0.051 (3)	0.052 (3)	0.043 (2)	0.004 (2)	−0.0077 (18)	0.0050 (18)
C35	0.043 (2)	0.049 (2)	0.0407 (19)	0.002 (2)	−0.0030 (17)	0.0088 (17)
N5	0.0358 (18)	0.048 (2)	0.0354 (15)	0.0004 (15)	−0.0022 (13)	0.0080 (14)
C36	0.037 (2)	0.054 (3)	0.044 (2)	0.006 (2)	−0.0011 (17)	0.0080 (19)
C37	0.045 (3)	0.062 (3)	0.048 (2)	0.011 (2)	−0.0038 (18)	0.004 (2)
C38	0.047 (3)	0.052 (3)	0.049 (2)	0.009 (2)	−0.0077 (19)	0.0036 (18)
C39	0.065 (3)	0.064 (3)	0.068 (3)	0.018 (3)	−0.009 (3)	0.015 (3)
S2	0.0454 (7)	0.0985 (11)	0.0666 (7)	0.0231 (7)	0.0050 (6)	0.0145 (7)
C40	0.042 (3)	0.130 (6)	0.067 (3)	0.006 (3)	−0.009 (2)	0.023 (3)
C41	0.039 (2)	0.100 (4)	0.051 (2)	−0.006 (3)	−0.009 (2)	0.006 (3)
C42	0.049 (3)	0.104 (4)	0.042 (2)	−0.012 (3)	−0.004 (2)	0.008 (3)
C43	0.051 (3)	0.090 (4)	0.041 (2)	−0.012 (3)	0.0000 (19)	0.001 (2)
C44	0.041 (2)	0.072 (3)	0.043 (2)	−0.015 (2)	−0.0008 (18)	−0.005 (2)
C45	0.046 (3)	0.088 (4)	0.055 (2)	−0.011 (3)	0.009 (2)	0.004 (3)
C46	0.038 (2)	0.099 (4)	0.061 (3)	−0.010 (3)	0.005 (2)	0.005 (3)
C47	0.052 (3)	0.059 (3)	0.0435 (19)	−0.020 (2)	0.013 (2)	−0.005 (2)
C48	0.047 (2)	0.042 (2)	0.043 (2)	−0.0123 (19)	0.0121 (17)	−0.0017 (16)
C49	0.069 (3)	0.043 (2)	0.052 (2)	−0.007 (2)	0.012 (2)	0.003 (2)
C50	0.110 (5)	0.043 (3)	0.067 (3)	−0.013 (3)	0.025 (3)	0.001 (2)
C51	0.106 (5)	0.053 (3)	0.071 (3)	−0.041 (4)	0.031 (3)	−0.009 (3)
C52	0.071 (3)	0.074 (4)	0.053 (2)	−0.038 (3)	0.013 (3)	−0.016 (3)
C53	0.047 (2)	0.037 (2)	0.0452 (19)	−0.0023 (18)	0.0059 (18)	0.0032 (16)
C54	0.060 (3)	0.042 (2)	0.046 (2)	−0.010 (2)	−0.002 (2)	0.0081 (19)
C55	0.064 (3)	0.044 (2)	0.057 (3)	−0.018 (2)	0.001 (2)	−0.002 (2)
C56	0.074 (3)	0.030 (2)	0.079 (3)	−0.012 (2)	0.008 (3)	0.005 (2)
C57	0.067 (3)	0.038 (2)	0.095 (4)	0.007 (2)	−0.007 (3)	0.021 (2)
C58	0.048 (3)	0.046 (3)	0.077 (3)	0.000 (2)	0.000 (2)	0.005 (2)
F1	0.075 (2)	0.0631 (18)	0.0844 (19)	−0.0207 (18)	−0.0285 (16)	0.0286 (16)
F2	0.082 (2)	0.0674 (19)	0.087 (2)	−0.0361 (17)	−0.0184 (19)	0.0069 (17)
F3	0.104 (3)	0.0368 (15)	0.118 (3)	−0.0149 (17)	0.003 (2)	0.0147 (16)
F4	0.097 (3)	0.057 (2)	0.176 (4)	0.007 (2)	−0.026 (3)	0.049 (2)
F5	0.0599 (19)	0.0634 (19)	0.125 (3)	−0.0026 (18)	−0.026 (2)	0.0207 (18)
C59	0.039 (2)	0.043 (2)	0.038 (2)	−0.0021 (17)	0.0040 (15)	−0.0020 (16)
C60	0.043 (2)	0.061 (3)	0.0383 (18)	0.001 (2)	−0.0013 (18)	−0.005 (2)
C61	0.038 (2)	0.052 (3)	0.051 (2)	0.004 (2)	0.0082 (18)	−0.0058 (19)
C62	0.051 (3)	0.046 (2)	0.041 (2)	−0.003 (2)	0.0152 (18)	−0.0072 (17)
C63	0.053 (3)	0.050 (2)	0.0338 (18)	−0.006 (2)	0.0047 (16)	−0.0030 (17)
C64	0.041 (2)	0.050 (2)	0.0411 (19)	−0.001 (2)	0.0033 (17)	−0.0011 (17)
F6	0.0516 (16)	0.110 (3)	0.0438 (13)	0.0159 (18)	−0.0031 (12)	−0.0095 (15)
F7	0.0442 (16)	0.096 (2)	0.0709 (17)	0.0195 (16)	0.0073 (13)	−0.0099 (16)
F8	0.0660 (18)	0.0676 (18)	0.0502 (14)	0.0031 (15)	0.0220 (13)	−0.0118 (12)
F9	0.0723 (19)	0.096 (2)	0.0375 (12)	−0.0010 (19)	−0.0001 (12)	−0.0108 (14)
F10	0.0475 (15)	0.084 (2)	0.0476 (13)	0.0108 (15)	−0.0008 (11)	−0.0029 (13)

### Geometric parameters (Å, º)

**Table:**

Ni1—N2	2.031 (3)	C32—H32	0.9500
Ni1—N4	2.036 (3)	C33—S1	1.834 (6)
Ni1—N3	2.036 (3)	C33—H33A	0.9900
Ni1—N1	2.041 (3)	C33—H33B	0.9900
Ni1—N5	2.106 (3)	S1—C34	1.782 (5)
N1—C1	1.368 (5)	C34—C35	1.387 (6)
N1—C4	1.373 (5)	C34—C38	1.401 (7)
N2—C6	1.371 (5)	C35—N5	1.347 (5)
N2—C9	1.373 (5)	C35—H35	0.9500
N3—C11	1.372 (5)	N5—C36	1.331 (5)
N3—C14	1.374 (5)	C36—C37	1.379 (6)
N4—C19	1.370 (5)	C36—H36	0.9500
N4—C16	1.371 (5)	C37—C38	1.403 (6)
C1—C20	1.402 (5)	C37—S2	1.772 (5)
C1—C2	1.427 (6)	C38—C39	1.505 (6)
C2—C3	1.352 (6)	C39—H39A	0.9800
C2—H2	0.9500	C39—H39B	0.9800
C3—C4	1.442 (5)	C39—H39C	0.9800
C3—H3	0.9500	S2—C40	1.832 (7)
C4—C5	1.386 (5)	C40—C41	1.501 (9)
C5—C6	1.400 (6)	C40—H40A	0.9900
C5—C21	1.498 (5)	C40—H40B	0.9900
C6—C7	1.435 (5)	C41—C42	1.379 (8)
C7—C8	1.344 (6)	C41—C46	1.388 (7)
C7—H7	0.9500	C42—C43	1.379 (8)
C8—C9	1.438 (6)	C42—H42	0.9500
C8—H8	0.9500	C43—C44	1.387 (7)
C9—C10	1.388 (6)	C43—H43	0.9500
C10—C11	1.396 (5)	C44—C45	1.386 (7)
C10—C53	1.501 (6)	C44—C47	1.488 (7)
C11—C12	1.439 (6)	C45—C46	1.386 (8)
C12—C13	1.352 (6)	C45—H45	0.9500
C12—H12	0.9500	C46—H46	0.9500
C13—C14	1.432 (6)	C47—C52	1.399 (6)
C13—H13	0.9500	C47—C48	1.404 (7)
C14—C15	1.390 (6)	C48—C49	1.394 (7)
C15—C16	1.397 (6)	C49—C50	1.388 (7)
C15—C59	1.493 (5)	C49—H49	0.9500
C16—C17	1.448 (5)	C50—C51	1.387 (10)
C17—C18	1.336 (6)	C50—H50	0.9500
C17—H17	0.9500	C51—C52	1.352 (9)
C18—C19	1.440 (5)	C51—H51	0.9500
C18—H18	0.9500	C52—H52	0.9500
C19—C20	1.396 (5)	C53—C54	1.382 (6)
C20—C48	1.489 (6)	C53—C58	1.385 (7)
C21—C26	1.396 (6)	C54—F1	1.334 (5)
C21—C22	1.397 (6)	C54—C55	1.379 (6)
C22—C23	1.394 (7)	C55—F2	1.345 (6)
C22—C27	1.496 (7)	C55—C56	1.348 (8)
C23—C24	1.374 (9)	C56—C57	1.344 (8)
C23—H23	0.9500	C56—F3	1.348 (5)
C24—C25	1.380 (8)	C57—F4	1.349 (6)
C24—H24	0.9500	C57—C58	1.379 (7)
C25—C26	1.378 (6)	C58—F5	1.335 (6)
C25—H25	0.9500	C59—C60	1.376 (6)
C26—H26	0.9500	C59—C64	1.386 (6)
C27—C28	1.381 (7)	C60—F6	1.351 (5)
C27—C32	1.395 (7)	C60—C61	1.376 (6)
C28—C29	1.392 (8)	C61—F7	1.336 (5)
C28—H28	0.9500	C61—C62	1.369 (6)
C29—C30	1.388 (8)	C62—F8	1.344 (5)
C29—H29	0.9500	C62—C63	1.370 (7)
C30—C31	1.391 (7)	C63—F9	1.341 (5)
C30—C33	1.490 (7)	C63—C64	1.375 (6)
C31—C32	1.368 (7)	C64—F10	1.343 (5)
C31—H31	0.9500		
			
N2—Ni1—N4	166.13 (12)	C30—C31—H31	119.2
N2—Ni1—N3	89.64 (13)	C31—C32—C27	120.8 (5)
N4—Ni1—N3	89.52 (12)	C31—C32—H32	119.6
N2—Ni1—N1	89.02 (12)	C27—C32—H32	119.6
N4—Ni1—N1	88.62 (12)	C30—C33—S1	112.3 (3)
N3—Ni1—N1	166.68 (13)	C30—C33—H33A	109.1
N2—Ni1—N5	95.43 (13)	S1—C33—H33A	109.1
N4—Ni1—N5	98.38 (13)	C30—C33—H33B	109.1
N3—Ni1—N5	88.44 (14)	S1—C33—H33B	109.1
N1—Ni1—N5	104.88 (13)	H33A—C33—H33B	107.9
C1—N1—C4	106.2 (3)	C34—S1—C33	100.7 (2)
C1—N1—Ni1	126.7 (2)	C35—C34—C38	119.9 (4)
C4—N1—Ni1	126.6 (2)	C35—C34—S1	118.1 (4)
C6—N2—C9	106.1 (3)	C38—C34—S1	122.0 (3)
C6—N2—Ni1	127.5 (3)	N5—C35—C34	123.0 (4)
C9—N2—Ni1	125.3 (3)	N5—C35—H35	118.5
C11—N3—C14	106.2 (3)	C34—C35—H35	118.5
C11—N3—Ni1	125.8 (2)	C36—N5—C35	117.1 (4)
C14—N3—Ni1	127.2 (3)	C36—N5—Ni1	119.5 (3)
C19—N4—C16	106.0 (3)	C35—N5—Ni1	121.4 (3)
C19—N4—Ni1	127.2 (2)	N5—C36—C37	124.0 (4)
C16—N4—Ni1	126.8 (3)	N5—C36—H36	118.0
N1—C1—C20	125.4 (4)	C37—C36—H36	118.0
N1—C1—C2	110.4 (3)	C36—C37—C38	119.7 (4)
C20—C1—C2	124.2 (4)	C36—C37—S2	118.1 (3)
C3—C2—C1	106.8 (4)	C38—C37—S2	122.2 (4)
C3—C2—H2	126.6	C34—C38—C37	116.3 (4)
C1—C2—H2	126.6	C34—C38—C39	121.9 (4)
C2—C3—C4	107.2 (4)	C37—C38—C39	121.7 (4)
C2—C3—H3	126.4	C38—C39—H39A	109.5
C4—C3—H3	126.4	C38—C39—H39B	109.5
N1—C4—C5	126.2 (3)	H39A—C39—H39B	109.5
N1—C4—C3	109.4 (3)	C38—C39—H39C	109.5
C5—C4—C3	124.4 (3)	H39A—C39—H39C	109.5
C4—C5—C6	124.8 (3)	H39B—C39—H39C	109.5
C4—C5—C21	115.4 (3)	C37—S2—C40	99.6 (3)
C6—C5—C21	119.7 (3)	C41—C40—S2	113.8 (4)
N2—C6—C5	125.1 (3)	C41—C40—H40A	108.8
N2—C6—C7	110.1 (4)	S2—C40—H40A	108.8
C5—C6—C7	124.7 (3)	C41—C40—H40B	108.8
C8—C7—C6	106.9 (3)	S2—C40—H40B	108.8
C8—C7—H7	126.6	H40A—C40—H40B	107.7
C6—C7—H7	126.6	C42—C41—C46	118.2 (6)
C7—C8—C9	107.5 (4)	C42—C41—C40	122.5 (5)
C7—C8—H8	126.2	C46—C41—C40	119.3 (5)
C9—C8—H8	126.2	C41—C42—C43	121.6 (5)
N2—C9—C10	125.0 (4)	C41—C42—H42	119.2
N2—C9—C8	109.5 (3)	C43—C42—H42	119.2
C10—C9—C8	125.5 (4)	C42—C43—C44	120.6 (5)
C9—C10—C11	126.7 (4)	C42—C43—H43	119.7
C9—C10—C53	117.3 (3)	C44—C43—H43	119.7
C11—C10—C53	116.1 (3)	C45—C44—C43	117.8 (5)
N3—C11—C10	124.1 (3)	C45—C44—C47	121.0 (4)
N3—C11—C12	109.9 (3)	C43—C44—C47	121.2 (5)
C10—C11—C12	126.0 (4)	C44—C45—C46	121.6 (5)
C13—C12—C11	106.7 (4)	C44—C45—H45	119.2
C13—C12—H12	126.7	C46—C45—H45	119.2
C11—C12—H12	126.7	C45—C46—C41	120.2 (5)
C12—C13—C14	107.5 (4)	C45—C46—H46	119.9
C12—C13—H13	126.2	C41—C46—H46	119.9
C14—C13—H13	126.2	C52—C47—C48	118.7 (5)
N3—C14—C15	124.9 (4)	C52—C47—C44	120.8 (5)
N3—C14—C13	109.6 (4)	C48—C47—C44	120.4 (4)
C15—C14—C13	125.4 (4)	C49—C48—C47	119.7 (4)
C14—C15—C16	125.7 (3)	C49—C48—C20	119.7 (4)
C14—C15—C59	117.7 (4)	C47—C48—C20	120.5 (4)
C16—C15—C59	116.6 (3)	C50—C49—C48	119.7 (6)
N4—C16—C15	125.5 (3)	C50—C49—H49	120.2
N4—C16—C17	109.7 (3)	C48—C49—H49	120.2
C15—C16—C17	124.7 (3)	C51—C50—C49	120.2 (6)
C18—C17—C16	107.0 (3)	C51—C50—H50	119.9
C18—C17—H17	126.5	C49—C50—H50	119.9
C16—C17—H17	126.5	C52—C51—C50	120.3 (5)
C17—C18—C19	107.4 (4)	C52—C51—H51	119.9
C17—C18—H18	126.3	C50—C51—H51	119.9
C19—C18—H18	126.3	C51—C52—C47	121.3 (5)
N4—C19—C20	125.5 (3)	C51—C52—H52	119.4
N4—C19—C18	109.9 (3)	C47—C52—H52	119.4
C20—C19—C18	124.6 (4)	C54—C53—C58	115.7 (4)
C19—C20—C1	124.5 (4)	C54—C53—C10	122.5 (4)
C19—C20—C48	117.8 (3)	C58—C53—C10	121.8 (4)
C1—C20—C48	117.6 (4)	F1—C54—C55	117.5 (4)
C26—C21—C22	119.9 (4)	F1—C54—C53	120.0 (4)
C26—C21—C5	119.2 (3)	C55—C54—C53	122.5 (4)
C22—C21—C5	120.7 (4)	F2—C55—C56	119.5 (4)
C23—C22—C21	118.4 (5)	F2—C55—C54	120.9 (5)
C23—C22—C27	121.8 (4)	C56—C55—C54	119.6 (5)
C21—C22—C27	119.8 (4)	C57—C56—F3	120.1 (5)
C24—C23—C22	121.2 (5)	C57—C56—C55	120.1 (4)
C24—C23—H23	119.4	F3—C56—C55	119.7 (5)
C22—C23—H23	119.4	C56—C57—F4	120.9 (5)
C23—C24—C25	120.2 (5)	C56—C57—C58	120.6 (5)
C23—C24—H24	119.9	F4—C57—C58	118.5 (5)
C25—C24—H24	119.9	F5—C58—C57	119.9 (5)
C26—C25—C24	119.8 (5)	F5—C58—C53	118.6 (4)
C26—C25—H25	120.1	C57—C58—C53	121.5 (5)
C24—C25—H25	120.1	C60—C59—C64	115.7 (4)
C25—C26—C21	120.5 (4)	C60—C59—C15	122.1 (4)
C25—C26—H26	119.8	C64—C59—C15	122.1 (4)
C21—C26—H26	119.8	F6—C60—C59	118.8 (4)
C28—C27—C32	118.3 (5)	F6—C60—C61	117.8 (4)
C28—C27—C22	123.1 (4)	C59—C60—C61	123.4 (4)
C32—C27—C22	118.4 (4)	F7—C61—C62	120.1 (4)
C27—C28—C29	120.4 (5)	F7—C61—C60	121.1 (4)
C27—C28—H28	119.8	C62—C61—C60	118.8 (4)
C29—C28—H28	119.8	F8—C62—C61	119.6 (4)
C30—C29—C28	121.3 (5)	F8—C62—C63	120.3 (4)
C30—C29—H29	119.4	C61—C62—C63	120.1 (4)
C28—C29—H29	119.4	F9—C63—C62	119.7 (4)
C29—C30—C31	117.4 (5)	F9—C63—C64	120.7 (4)
C29—C30—C33	122.0 (5)	C62—C63—C64	119.6 (4)
C31—C30—C33	120.6 (5)	F10—C64—C63	118.3 (4)
C32—C31—C30	121.6 (5)	F10—C64—C59	119.3 (4)
C32—C31—H31	119.2	C63—C64—C59	122.3 (4)

### Hydrogen-bond geometry (Å, º)

**Table:**

*D*—H···*A*	*D*—H	H···*A*	*D*···*A*	*D*—H···*A*
C33—H33*B*···F8^i^	0.99	2.63	3.592 (6)	164
C35—H35···N2	0.95	2.58	3.125 (5)	117
C36—H36···N4	0.95	2.60	3.206 (5)	122

Symmetry code: (i) −*x*+3/2, −*y*+1, *z*+1/2.
